# The risk of tuberculosis disease in rheumatoid arthritis patients on biologics and targeted therapy: A 15-year real world experience in Taiwan

**DOI:** 10.1371/journal.pone.0178035

**Published:** 2017-06-01

**Authors:** Chong Hong Lim, Hsin-Hua Chen, Yi-Hsing Chen, Der-Yuan Chen, Wen-Nan Huang, Jaw-Ji Tsai, Tsu-Yi Hsieh, Chia-Wei Hsieh, Wei-Ting Hung, Ching-Tsai Lin, Kuo-Lung Lai, Kuo-Tung Tang, Chih-Wei Tseng, Yi-Ming Chen

**Affiliations:** 1 Rheumatology Unit, Department of Internal Medicine, Pulau Pinang General Hospital, Georgetown, Malaysia; 2 Division of Allergy, Immunology and Rheumatology, Department of Internal Medicine, Taichung Veterans General Hospital, Taichung, Taiwan; 3 Department of Medical Education and Research, Taichung Veterans General Hospital, Taichung, Taiwan; 4 School of Medicine, National Yang-Ming University, Taipei, Taiwan; 5 Rong Hsing Research Center for Translational Medicine, National Chung Hsing University, Taichung, Taiwan; 6 Institute of Biomedical Science and Rong Hsing Research Center for Translational Medicine, Chung-Hsing University, Taichung, Taiwan; 7 School of Medicine, Chung-Shan Medical University, Taichung, Taiwan; Hospital Universitari de Bellvitge, SPAIN

## Abstract

The objective of this study is to determine the risk of tuberculosis (TB) disease in biologics users among rheumatoid arthritis (RA) patients in Taiwan from 2000 to 2015. This retrospective cohort study enrolled adult RA patients initiated on first biologics at Taichung Veterans General Hospital. TB risks were determined as hazard ratio (HR) with 95% confidence interval (CI) using cox regression. A total of 951 patients were recruited; etanercept (n = 443), adalimumab (n = 332), abatacept (n = 74), golimumab (n = 60), tocilizumab (n = 31) and tofacitinib (n = 11). Twenty-four TB cases were identified; 13 in etanercept and 11 in adalimumab group with the TB incidence rate of 889.3/ 100,000 and 1055.6/ 100,000 patient-years respectively. There was no significant difference in TB risk between adalimumab and etanercept users with an incidence rate ratio of 1.27 (*p* = 0.556 by Poisson model). Significant 2-year TB risk factors included elderly patient >65 year-old (HR: 2.72, 95% CI: 1.06–6.99, *p* = 0.037), history of TB (HR: 6.24, 95% CI: 1.77–22.00, *p* = 0.004) and daily glucocorticoid use ≥5mg (HR:5.01, 95% CI: 1.46–17.21, *p* = 0.010). Sulfasalazine treatment appeared to be protective (HR: 0.32, 95% CI: 0.11–0.97, *p* = 0.043). Risk management plan (RMP) for TB before initiation of biologics commenced in 2012. The 2-year TB risks after RMP was compared with that before 2012 (HR:0.67, 95% CI: 0.30–1.49, *p* = 0.323). Elderly RA patients with a history of previous TB infection and concomitant moderate dose glucocorticoid were at higher risk of TB disease. Concurrent sulfasalazine treatment appeared to be a protective factor against TB disease.

## Introduction

Tuberculosis (TB) remains one of the top 10 leading cause of death worldwide. It was estimated to have 10.4 million new cases worldwide in 2015 [1]. In Taiwan, TB is still prevalent leading to an intermediate healthcare burden. Incidence of TB was reported as 45.6 cases per 100,000 populations with 10,534 new cases in 2015 [2].

As shown in a Spanish study, rheumatoid arthritis (RA) patient has 4-fold increased risk of TB disease as compared to general population [3]. It is well established that Th1 mediated cytokines play crucial roles in the protective immunity against TB disease [4–6]. With the introduction of biological disease modifying anti-rheumatic drugs (bDMARDs) and targeted disease modifying anti-rheumatic drugs (tDMARDs) that act against the host defense immunities, the risk of TB disease is further increased among bDMARDs and tDMARDs users in RA patients. However, the risk of TB disease varied in different bDMARDs and tDMARDs. In tumor necrosis factor-α inhibitor (TNFi), TB risk was reported to be higher in monoclonal antibody TNFi than in soluble receptor for TNF-α [7–10]. For non-TNFi bDMARDs such as tocilizumab (interleukin-6 inhibitor) and abatacept (T-cell costimulator blocker) the TB risk was reported to be relatively low [11, 12]. For tofacitinib (janus kinase inhibitor) the TB rate varied according to different regional backgrounds of TB endemicity [13].

In Taiwan, the available bDMARDs and tDMARDs include etanercept, adalimumab, golimumab, tocilizumab, abatacept, rituximab and tofacitinib. The risk of TB disease in RA was reported to be 2.28 times higher than the general population [14]. Previous population-based studies showed a 2.67–4.87 times increased in TB risk among TNFi users in RA patients [15, 16]. However, the TB risk of newer TNFi, golimumab, non-TNFi bDMARDs, and tDMARDs has not yet been investigated. Therefore, we aimed to determine the risk of TB disease in bDMARDs and tDMARDs users among RA patients in Taiwan from 2000 to 2015.

## Materials and methods

### Data source

The data of the study was obtained by using the Hyperion Enterprise Performance Management System (Oracle, USA) from Taichung Veterans General Hospital, a tertiary rheumatology center in central Taiwan. This study was conducted in compliance with the Helsinki Declaration. The approval by the Institution Review Board of Taichung Veterans General Hospital was acquired (CE14149B-1). The study was analyzed anonymously, therefore, no informed consent was obtained from the participants.

### Study cohort

This retrospective cohort study enrolled all adult (≧18-year-old) inpatients and outpatients initiated on the first bDMARDs or tDMARDs, with a diagnosis of RA from 1^st^ January 2000 to 31^st^ August 2015. RA patients were identified by using the International Classification of Diseases, 9th Revision, Clinical Modification (ICD-9-CM) code 714.0. The bDMARDs and tDMARDs studied were etanercept, adalimumab, golimumab, tocilizumab, abatacept, and tofacitinib. Rituximab was not included because it was approved as second-line bDMARDs in Taiwan. Comorbidities such as diabetes mellitus (DM) and cardiovascular disease (CVD) were detected by the ICD-9-CM code (DM: 250.x and CVD: 410–414) and the prescription of medications for DM and CVD. Chronic kidney disease (serum creatinine >1.5 mg/dl), hepatitis (serum glutamic pyruvic transaminase > 40 U/l) and anemia (hemoglobin level < 12 g/dl) were also identified upon the initiation of bDMARDs or tDMARDs. We excluded patients who had a concomitant diagnosis of psoriatic arthritis, spondyloarthritis, inflammatory bowel diseases or Behcet’s disease. Patients who had used bDMARDs or tDMARDs prior to 1^st^ January 2000 were also excluded from the study. Subsequent bDMARDs or tDMARDs treatment was not included for analysis. Concurrent medications used including conventional synthetic DMARDs (csDMARDs) and steroid were identified. Laboratory investigations such as hemoglobin level (g/dl), creatinine level (mg/dl) and glutamic pyruvic transaminase (U/l) upon the initiation of bDMARDs or tDMARDs were also recorded.

### Latent TB screening

In Taiwan, the latent TB screening and treatment policy before initiation of biologics commenced in 2012 [17]. As per TB risk management plan (RMP), every patient must undergo TB screening before initiation of biologics. Since BCG is universally vaccinated in Taiwan, latent TB was determined by QuantiFERON-TB Gold (QFT-G) assays as per our local guideline [17]. The result of the QFT-G assay was defined as positive for latent TB if the interferon-γ (IFN-γ) level≧ 0.35 IU/ml in TB-specific antigens-stimulated wells after subtracting the level of the nil well as per manufacturer’s instructions (Cellestis Ltd., Victoria, Australia).

### Study outcome

The primary outcome of this study was the manifestation of TB disease during the first prescribed bDMARDs or tDMARDs prior to drug switching or the end of this study. In our study, TB was defined based on the ICD-9-CM (010–018), and the prescription of 2 or more anti-TB drugs within 6 months of the TB diagnosis made. The anti-TB drugs used were isoniazid, ethambutol, rifampin, pyrazinamide, prothionamide, kanamycin, streptomycin, amikacin, ciprofloxacin, ofloxacin, levofloxacin, moxifloxacin, clarithromycin and thioridazine. This method to identify TB has been validated earlier [18]. All cases of TB diseases were proved by positive culture or pathological findings of tissue biopsy. History of TB referred to the occurrence of TB prior to the commencement of bDMARDs or tDMARDs. All patients were censored till the occurrence of TB disease, lost to follow-up in the hospital database due to death or relocation to another institute or the end of observation, whichever came first.

### Statistical analysis

The demographic data of continuous variables was shown as a mean ± standard deviation; and for categorical variables as a number of patients. An analysis of variance (ANOVA) test was used to examine the unadjusted comparison. The TB incidence rate (IR) per 100,000 patients was calculated by dividing the new TB cases by the total case patient-year. Comparisons of incidence rate ratio (IRR) were also determined using Poisson model. The cumulative survival rates of TB disease among bDMARDs or tDMARDs were calculated using Kaplan-Meier method and compared by log-rank test. Potential risk factors for TB disease were determined by using multivariable cox regression. A 2-year TB risk was calculated by censoring subjects at 2 years after initiation of bDMARDs or tDMARDs. Significant risk factors were calculated as hazard ratio (HR) with its 95% confidence interval (CI). All data was analyzed using the Statistical Package for the Social Sciences (SPSS) version 22.0. Significant value was set at *p* < 0.05.

## Results

### Demographic data

There was a total of 951 patients recruited in this cohort (Table 1, S1 Dataset). Majority of the patients (81.5%) were etanercept (n = 443) and adalimumab (n = 332) users followed by abatacept (n = 74), golimumab (n = 60), tocilizumab (n = 31) and tofacitinib (n = 11). The mean age of the patients was 52.6 ± 13.8 years, and the mean duration of disease was 8.1 ± 4.6 years. Of the 951 patients, 81% were female. The mean duration of follow-up after initiation of bDMARDs and tsDMARDs was 2.9 ± 2.5 years. Co-morbidities of diabetes mellitus (2.5%), cardiovascular disease (2.6%), chronic kidney disease (5.0%), hepatitis (8.0%) and anemia (63.4%) were observed in this cohort. There were 28 (2.9%) patients with a history of TB prior to starting bDMARDs/tDMARDs; 10 (35.7%) of them were treated for TB from available hospital database. The most common csDMARDs used concomitantly was methotrexate (68.6%). There were 829 (87.2%) patients used glucocorticoid while on bDMARDs or tDMARDs therapies.

**Table 1:** Demographic data and clinical characteristics among RA patients initiating bDMARDs and tDMARDs.

	Total	ETN	ADA	GLN	TCZ	ABA	TOF	p-value
	(n = 951)	(n = 443)	(n = 332)	(n = 60)	(n = 31)	(n = 74)	(n = 11)	
	n (%)	n (%)	n (%)	n (%)	n (%)	n (%)	n (%)	
**Age (mean ± SD)**	52.6±13.8	52.8±14.5	51.8±13.4	48.8±13.0	54.4±12.8	56.7±13.2	57.1±13.5	0.016
**Age group**								0.015
<65 years	772 (81.2)	347(78.3)	283 (85.2)	54 (90.0)	25 (80.6)	53 (71.6)	10 (90.9)	
≥65 years	179 (18.8)	96 (21.7)	49 (14.8)	6 (10.0)	6 (19.4)	21 (28.4)	1 (9.1)	
**Gender**								0.032
Female	770 (81.0)	347(78.3)	281 (84.6)	52 (86.7)	23 (74.2)	61 (82.4)	6 (54.5)	
**Disease duration, years (mean ± SD)**	8.1±4.6	8.1±4.4	8.7±4.7	6.3±4.7	6.8±5.0	7.6±4.5	5.8±6.0	<0.001
**Follow-up duration, years (mean ± SD)**	2.9±2.5	3.3±2.6	3.2±2.5	1.6±1.1	1.8±1.1	1.4±0.9	0.2±0.2	<0.001
**Comorbidities**								
DM	24 (2.5)	10 (2.3)	5 (1.5)	1 (1.7)	2 (6.5)	5(6.8)	1 (9.1)	0.052
CVD	25 (2.6)	6 (1.4)	13 (3.9)	0 (0)	1 (3.2)	4 (5.4)	1 (9.1)	0.003
CKD	48 (5.0)	29 (6.5)	12 (3.6)	3 (5.0)	2 (6.5)	2 (2.7)	0 (0)	0.409
Hepatitis	74 (8.0)	30 (7.2)	28 (8.4)	2 (3.3)	5 (16.1)	8 (10.8)	1 (9.1)	0.319
Anemia	581 (63.4)	269 (64.0)	210 (65.0)	40 (67.8)	12 (40.0)	43 (58.9)	7 (63.6)	0.124
**TB history**	28 (2.9)	15 (3.4)	6 (1.8)	1 (1.7)	1 (3.2)	4 (5.4)	1 (9.1)	0.394
**Concomitant DMARD**								
Methotrexate	652 (68.6)	318 (71.8)	222 (66.9)	40 (66.7)	23 (74.2)	42 (56.8)	7 (63.6)	0.154
Leflunomide	153 (16.1)	64(14.4)	43 (13.0)	19 (31.7)	5 (16.1)	21 (28.4)	1(9.1)	<0.001
Sulfasalazine	370 (38.9)	221(49.9)	99 (29.8)	23 (38.3)	5 (16.1)	22 (29.7)	0 (0.0)	<0.001
Hydroxychloroquine	546 (57.4)	275 (62.1)	155 (46.7)	39 (65.0)	12 (38.7)	58 (78.4)	7 (63.6)	<0.001
Ciclosporin	105 (11.0)	52 (11.7)	30 (9.0)	8 (13.3)	3 (9.7)	11 (14.9)	1 (9.1)	0.684
Azathioprine	32 (3.4)	5 (1.1)	17 (5.1)	4 (6.7)	1 (3.2)	5 (6.8)	0 (0.0)	0.010
**Steroid**	829 (87.2)	384 (86.7)	290 (87.3)	55 (91.7)	28 (90.3)	65 (87.8)	7 (63.6)	0.225
**Initiation of bDMARDs/tDMARDs**								<0.001
Before 2012	571 (60.0)	335 (75.6)	236 (71.1)	0 (0)	0 (0)	0 (0)	0 (0)	
After 2012	380 (40.0)	108 (24.4)	96 (28.9)	60 (100)	31 (100)	74 (100)	11 (100)	

Abbreviations: RA, rheumatoid arthritis; bDMARDs, biological drug modifying anti-rheumatic drugs; tDMARDs, targeted disease modifying anti-rheumatic drugs, ETN, etanercept; ADA, adalimumab; GLN, golimumab; TCZ, tocilizumab; ABA, abatacept; TOF, tofacitinib; DM, diabetes mellitus; CVD, cardiovascular disease; CKD, chronic kidney disease; TB, tuberculosis.

### Incidence of TB among bDMARDs and tDMARDs users

**Fig 1:**
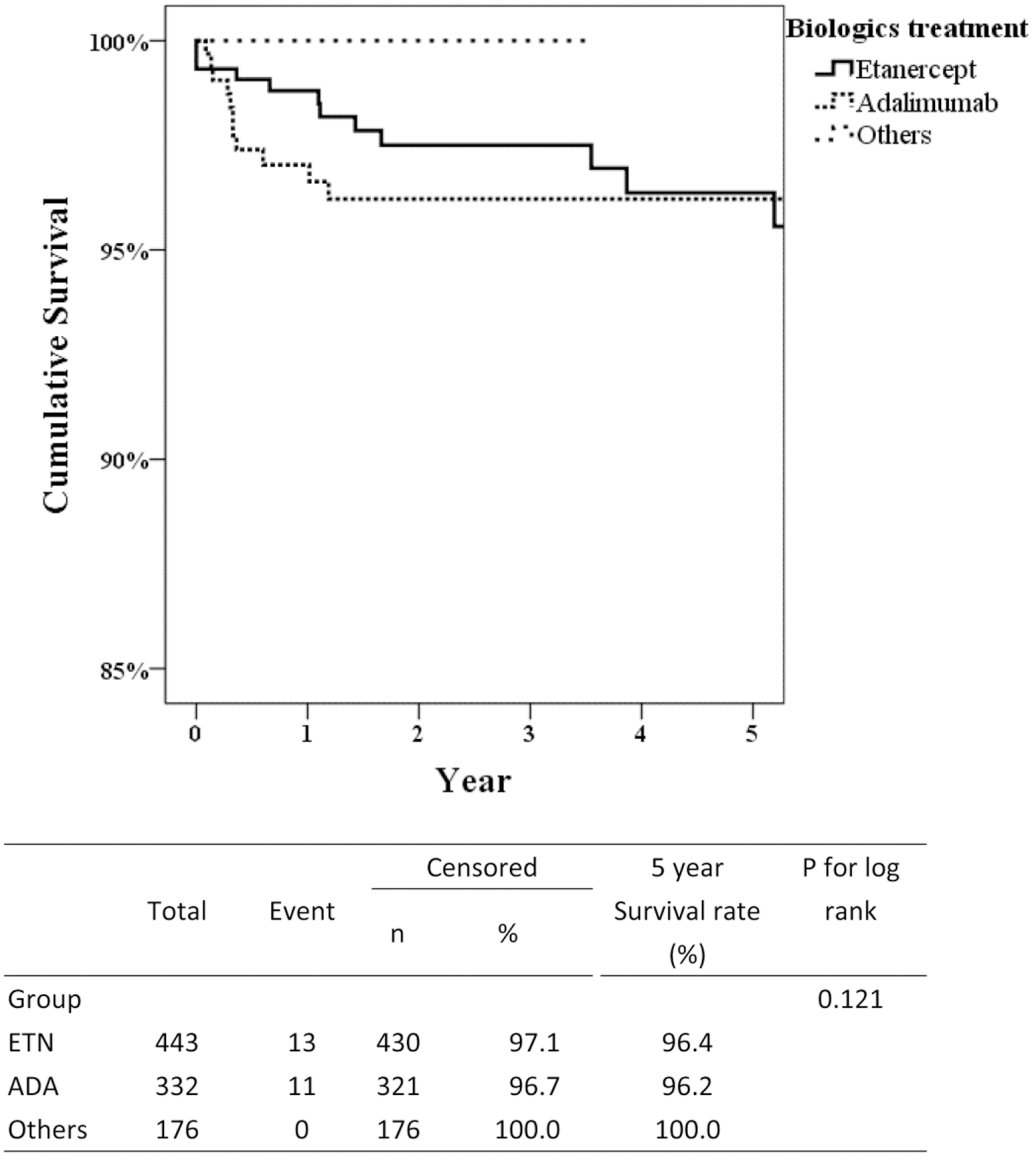
Kaplan-Meier plot of cumulative survival in TB disease among bDMARDs and tDMARDs treatment. Others group includes golimumab, tocilizumab, abatacept and tofacitinib. P-value was determined by the log-rank test. Abrreviations: TB, tuberculosis; bDMARDS, biological drug modifying anti-rheumatic drugs; tDMARDs, targeted disease modifying anti-rheumatic drugs; ETN: etanercept; ADA: adalimumab.

A total of 24 cases of TB were identified throughout the observation period (Table 2). Eleven TB cases were found in the adalimumab group and 13 cases in the etanercept group (Table 3). The TB IR was 889.3/ 10^5^ years and 1055.6/ 10^5^ years for etanercept and adalimumab groups respectively. There were no TB cases observed in the golimumab and non-TNFi groups. In Taiwan, the latent TB RMP before initiation of biologics commenced in 2012. The TB IR was comparable before and after RMP (866.6/ 10^5^ and 879.9/ 10^5^ years, respectively). However, the IRRs for adalimumab before and after nationwide RMP were 1.35 (95% CI:0.73–2.48, *p* = 0.334 by Poisson model) and 1.09 (95% CI:0.55–2.19, *p* = 0.803 by Poisson model), respectively (Table 2). In addition, we further analyzed the IRR after exclusion subjects with TB history in S1 Table. Interestingly, the TB IRR for adalimumab was 1.87 (95% CI:1.27–2.73, *p* = 0.001 by Poisson model) in patients without TB infection history but 1.27 (95% CI: 0.76–2.13, *p* = 0.556 by Poisson model) in all subjects with etanercept as a reference. Compared with the etanercept group, adalimumab users had a s shorter disease duration (5.0 ± 4.0 vs. 9.0 ± 3.9 years, *p* = 0.04, Table 3). Moreover, the majority of TB cases occurred within 1 year of adalimumab treatment compared with etanercept (81.8% vs. 38.5%, *p* = 0.047). Approximately 70% of TB cases were pulmonary TB. The cumulative survival rate of TB disease at 5 years was 100% for golimumab, non-TNFi bDMARDs, and tDMARDs (Fig 1). Although in the first 3 years of bDMARDs therapy, the survival rates were numerically better in etanercept treatment compared with adalimumab treatment, the 5-year cumulative data was indistinguishable (96.4% vs. 96.2%, *p* = 0.121).

**Table 2:** Incidence of TB according to bDMARDs and stratified before and after 2012.

	Total	Event (%)	Total person-years	Incidence Rate (/10^5^ years)	IRR (95% CI) †
**2000–2011**					
**ETN**	335	10 (3.0)	1286.4	777.4	1
**ADA**	236	9 (3.8)	906.0	993.4	1.35 (0.73–2.48)*
**GLN**	-	-	-	-	-
**TCZ**	-	-	-	-	-
**ABA**	-	-	-	-	-
**TOF**	-	-	-	-	-
**Total**	571	19(3.3)	2192.4	866.6	-
**2012–2015**					
**ETN**	108	3 (2.8)	175.4	1710.6	1
**ADA**	96	2(2.1)	136.1	1469.1	1.09 (0.55–2.19)^#^
**GLN**	60	0 (0.0)	94	0.0	-
**TCZ**	31	0 (0.0)	55.49	0.0	-
**ABA**	74	0 (0.0)	105.3	0.0	-
**TOF**	11	0 (0.0)	1.91	0.0	-
**Total**	380	5(1.3)	568.2	879.9	-
**2000–2015**					
**ETN**	443	13 (2.9)	1461.8	889.3	1
**ADA**	332	11 (3.3)	1042.1	1055.6	1.27 (0.76–2.13)^&^
**GLN**	60	0 (0.0)	94.0	0	-
**TCZ**	31	0 (0.0)	55.5	0	-
**ABA**	74	0 (0.0)	105.3	0	-
**TOF**	11	0 (0.0)	1.9	0	-
**Total**	951	24(2.5)	2758.7	870.0	-

^†^Adjusted for sex and age;

*p = 0.334;

^#^*p* = 0.803;

^&^*p* = 0.556 by Poisson model

Abbreviations: TB, tuberculosis; bDMARDs, biological drug modifying anti-rheumatic drugs; tDMARDs, targeted disease modifying anti-rheumatic drugs; ETN, etanercept; ADA, adalimumab; GLN, golimumab; TCZ, tocilizumab; ABA, abatacept; TOF, tofacitinib; IRR, incidence rate ratio.

**Table 3:** Comparison of etanercept and adalimumab treated RA patients with TB disease.

	Etanercept (N = 13)	Adalimumab (N = 11)	P-value
**Age, years**	58.1±9.7	65.0±9.3	0.124
≥65 year of age	23.1	45.5	0.390
**Gender (Female)**	76.9	81.8	1.000
**Disease duration, years**	9.0±3.9	5.0±4.0	0.040
≥4 years	100	54.5	0.011
**TB history**	30.8	0	0.098
**Latent TB**	38.5	36.4	1.000
**Average time to TB disease (days)**	1019±840	399±827	0.006
≤1 year	38.5	81.8	0.047
**Pulmonary TB**	69.2	72.7	0.854
**Extra-pulmonary TB**	30.8	27.3	0.854
**DM**	7.7	0	1.000
**CKD**	7.7	0	1.000
**Methotrexate**	84.6	90.9	1.000
**Hydroxychloroquine**	46.2	27.3	0.423
**Initiation of bDMARDs/ tDMARDs**			1.000
Before 2012	76.9	81.8	
After 2012	23.1	18.2	

Data presented as mean ± SD or percent

Abbreviations: RA, rheumatoid arthritis; TB, tuberculosis; bDMARDs, biological drug modifying anti-rheumatic drugs; tDMARDs, targeted disease modifying anti-rheumatic drugs; DM, diabetes mellitus; CKD, chronic kidney disease.

### Two-year risks of TB disease

Because the risk of TB infection is higher in the first years, we calculated the 2-year TB infection risk. In the multivariable analyses for 2-year TB risk among bDMARDs users (Table 4), significant risk factors identified were elderly age more than 65 year-old (HR: 2.72, 95% CI: 1.06–6.99, *p* = 0.037), history of TB (HR: 6.24, 95% CI: 1.77–22.00, *p* = 0.004), and concomitant steroid use of more than 5mg/ day (OR: 5.01, 95% CI: 1.46–17.21, *p* = 0.010). The use of concomitant sulfasalazine appeared to be a protective factor against TB disease among bDMARDs and tDMARDs users (HR: 0.32, 95% CI: 0.11–0.97, *p* = 0.043).

**Table 4:** Multivariable analyses for 2-year TB risk in bDMARDs users.

	Univariable			Multivariable		
	HR	95%CI	P-value	HR	95%CI	P-value
**Age**						
<65 year of age	Reference	Reference		Reference	Reference	
≥65 year of age	3.24	1.46–7.23	0.004	2.72	1.06–6.99	0.037
**Sex**						
Women	Reference	Reference				
Men	0.90	0.41–1.97	0.791			
**Disease duration**						
≥4 years	Reference	Reference				
<4 years	1.20	0.50–2.84	0.683			
**TB history**	5.31	1.60–17.63	0.006	6.24	1.77–22.00	0.004
**Latent TB**	1.66	0.73–3.79	0.229			
**bDMARDs**						
Etanercept	Reference	Reference				
Adalimumab	1.41	0.66–3.02	0.371			
Golimumab	-	-	-			
Tocilizumab	-	-	-			
Abatacept	-	-	-			
Tofacitinib	-	-	-			
**Diabetes mellitus**	2.98	0.71–12.57	0.138			
**CVD**	1.20	0.16–8.81	0.861			
**CKD**	1.81	0.43–7.66	0.418			
**Hepatitis**	1.22	0.37–4.05	0.749			
**Anemia**	2.01	0.87–4.65	0.105			
**Concomitant medications**						
Methotrexate	1.88	0.82–4.30	0.133			
Leflunomide	0.74	0.22–2.44	0.616			
Sulfasalazine	0.26	0.09–0.75	0.013	0.32	0.11–0.97	0.043
Hydroxychloroquine	0.58	0.25–1.37	0.215			
Ciclosporine	1.37	0.48–3.97	0.558			
Corticosteroid						
<5mg	Reference	Reference		Reference	Reference	
≥5mg	5.02	1.47–17.16	0.010	5.01	1.46–17.21	0.010
**Initiation of bDMARDs/tDMARDs**						
Before 2012	Reference	Reference				
After 2012	0.67	0.30–1.49	0.323			

Abrreviations: OR, odds ratio, TB, tuberculosis; bDMARDS, biological drug modifying anti-rheumatic drugs; tDMARDs, targeted disease modifying anti-rheumatic drugs; CVD, cardiovascular disease; CKD, chronic kidney disease

## Discussions

This hospital-based, retrospective cohort study showed a higher TB risk for etanercept and adalimumab users but not for golimumab or non-TNF inhibitors/ tDMARDs. We also found that elderly RA patients with a history of TB, and those who consumed moderate dose of glucocorticoid were at a higher risk of TB disease. This result may illuminate the treatment strategy of low-risk bDMARDs for high-risk patients.

In this 15-year data from the year of 2000 to 2015, the TB IR for etanercept and adalimumab was found to be lower when compared to a population-based study done from 1999 to 2009 [16]. The reduction in TB IR might be due to increased awareness by the rheumatologists in detecting, screening and treating for TB as well as latent TB prior to starting bDMARDs. In Taiwan, etanercept was introduced before the year 2000 but adalimumab was not made available until 2007. Since 2012, the Taiwan Rheumatology Association had implemented a RMP for bDMARDs users [17]. The recommendations in RMP for TB and latent TB screening include a detailed history and physical examination, chest x-ray, sputum, tuberculin skin test, and QFT-G assays. With these, TB/ latent TB screening before commencing and during bDMARDs therapy will be consolidated. Although our data showed comparable IR for TB before and after RMP, the 2-year TB risk was numerically lower after 2012 compared with that before 2012 (HR:0.67, 95% CI: 0.30–1.49, p = 0.323). The Taiwan RMP for TB was essentially based on studies from the same hospital of this study [17, 19]. Similar IR before and after 2012 could be partly explained by limited sample size, short follow-up duration and early implantation of RMP in this institute.

Previous data also reported an increased risk of TB disease in adalimumab users when compared to etanercept users, with an IRR of 2.35 during the years 1999 to 2009. [16]. Our initial analysis of year 2000–2015 did not show similar finding. We hypothesized that it could be related to the patient selection and strict implementation of RMP after 2012. We further analyzed the IRR after exclusion those with TB history in S1 Table. Interestingly, the TB IRR for adalimumab was significantly elevated after exclusion patients with TB infection history. In Tables 3 and 4 cases with TB history received etanercept therapy. It is well-known that TB history is a major risk for TB reactivation after anti-TNF-α inhibitors treatment. The relatively lower IRR for adalimumab in all enrolled subjects might be explained by the physicians’ judgement for using more etanercept in high-risk patients with history of TB infection, reducing the risk differences in etanercept and adalimumab. Moreover, during the 15-year observation period, patient characteristics, comorbidities and disease severities might have changed over time. To minimized the influences in these potential confounding factors, we further stratified the observation period before and after 2012. The IRR for adalimumab decreased substantially after RMP.

Patients with a history of previous TB and latent TB disease had a significantly higher risk of TB disease up to 14.59-fold and 2.58-fold respectively. This observation was in agreement with the findings in previous studies [20, 21]. The majority of adalimumab patients (81.8%) contracted TB within 1 year of bDMARDs initiation. For the etanercept group, TB was detected in 5 patients (38.5%) within 1 year of starting bDMARDs. These findings were in parallel with our previous study showing a biphasic pattern of TB disease among bDMARDs users; the early emergence of TB in adalimumab users is likely to be caused by a latent TB re-activation, and late TB emergence would be likely due to a new TB infection [19]. In this cohort, TB disease in the adalimumab group might be due to the re-activation of latent TB while the majority of TB disease in the etanercept group was possibly owing to a new TB infection.

The use of csDMARDs was reported to increase the risk of TB disease [22–24]. In this study, the analysis revealed that RA patients who concomitantly used methotrexate but not leflunomide with bDMARDs had 3.97-fold increased TB risk (S2 Table). This finding was in agreement with a systemic review which explained the additional TB risk was related to the synergistic effect of TNFi and methotrexate rather than the intrinsic TB risk of methotrexate alone [24]. On the contrary, the findings in a study done by Brode *et al*. showed vice versa [22]. Interestingly, our cohort also demonstrated that RA patients who administered hydroxychloroquine concomitantly with bDMARDs had a significantly lower risk of contracting TB disease (S2 Table). This could be possibly due to the pharmacological property of hydroxychloroquine that has a lysosomotropic effect [25], which is an important inhibitory effect in the immunology of TB disease [26]. This observation was compatible with the findings by Brode *et al*. [22]. A Higher dose of steroid use (prednisolone equivalent dose ≥ 5mg/ day) increased 5-fold risk of TB disease observed in this study. The result was in parallel to our previous population study and a Canadian cohort [27, 28]. Other factors associated with increased TB risk such as diabetes mellitus, chronic kidney disease, and the use of ciclosporin were reported in previous studies [20, 22, 23, 29]. However, these associations were not observed in this cohort. Possibly this could be explained by the patient selection criteria in this study and the outcome of the RMP. The risk of TB infection in our study is increased during the first years but decreased afterward. We further investigated the 2-year TB risk by censoring our cases after 2 years. We found that concomitant sulfasalazine treatment was associated with reduced TB infection risk. This novel finding has never been reported in the literature. Sulfasalazine is the prodrug of sulphapyridine and 5-aminosalicylic acid. It might be plausible that the anti-bacterial effects of sulphonamides could also reduce TB infection risk. Further study is needed to confirm our findings.

In this cohort, there was no TB detected in patients who used golimumab, tocilizumab, abatacept or tofacitinib. Compared to a golimumab long-term extension randomized controlled study, the analysis did not definitively show an increase in TB disease during the 3-year follow-up period [30]. For tocilizumab, a post-marketing surveillance of 7,901 RA patients, a low TB IR was reported (0.05%) [31]. In a 5-year extension study of abatacept among RA patients, no TB was observed [32]. In comparison to a clinical trial done by Winthrop *et al*., the crude TB IR for tofacitinib was 0.75% in a region with a background of high TB prevalence [13]. Although the cumulative number of RA patients administrating golimumab, non-TNFi and tofacitinib in this study was still low and the duration of recruitment for tofacitinib was short, our results demonstrated that TB risk could remain low if RA patients were screened and treated vigilantly for TB/ latent TB prior to starting bDMARDs/ tDMARDs.

To the best of our knowledge, this is the first study performed in Taiwan which has examined the risk of TB in newer bDMARDs/ tDMARDs including golimumab, tocilizumab, abatacept and tofacitinib. This may be an additional merit as this study was able to demonstrate the real-world experience of TB risk among bDMARDs/ tDMARDs users within the background of an intermediate TB burden, as compared to the majority of previous reports which were from regions where TB prevalence is low. However, several limitations were encountered in this study. First, in the real-world observational study, confounded by indication cannot be completely avoided. Patients receiving golimumab appeared to be younger with more female and shorter disease duration. Old age is a known risk factor for TB disease. In addition, Etanercept-treated patients were taking more hydroxychloroquine and sulfasalazine than adalimumab-treated patients. Our results also discovered hydroxychloroquine and sulfasalazine to be protective against TB disease. Although age, disease duration, hydroxychloroquine and sulfasalazine were adjusted in the Cox model, these inherent differences in patient characteristics might still underestimate the risk of TB disease in golimumab and etanercept-treated patients.

Secondly, miscoding or misclassification of the diagnoses may have occurred. The bias in the diagnosis of RA patients who used bDMARDs or tDMARDs should be minimal as these patients were obliged to fulfill the RA classification criteria by American College of Rheumatology (ACR) 1987 or the ACR/ European League Against Rheumatism (EULAR) 2010. Furthermore, a careful examination by experienced rheumatologists which included patient’s medical history, imaging, and laboratory data was also required before the bDMARDs or tDMARDs reimbursement by Taiwan’s National Health Insurance program. Self-paid bDMARDs or tDMARDs users may be present however the number of these patients would not have impacted the results. Thirdly, this retrospective data did not provide complete information on TB risk factors. Important confounding factors such as socio-economic status, RA disease activity, smoking status and body weight that may be potential risks to TB disease were not recorded. However, Taiwan National Health Insurance only reimbursed bDMARDs and tDMARDs treatment for RA patients with high disease activity, which in turn minimized the potential confounding effect of the disease severity. Lastly, newer bDMARDs and tDMARDs such as golimumab, tocilizumab and abatacept were introduced in Taiwan in the year 2012 while tofacitinib in the year 2015. The data obtained in this cohort might not represent its actual characteristic because both the duration of recruitment for these bDMARDs was short, and the cumulative number of patients was small. However, this study only enrolled RA patients with first bDMARDs or tDMARDs treatment. The exclusion of bDMARDs or tDMARDs-experienced RA patients also reduces the possible interference of prolonged immunosuppression by previous treatment. A real-world study of more enrolled patients and long-term follow-up is needed to determine the TB risk in newer bDMARDs and tofacitinib.

In conclusion, TB risks were found to be varying in bDMARDs, with etanercept and adalimumab demonstrating similarly increased risks, while relatively low risks were seen in newer bDMARDs and tofacitinib. The identification of different risk treatment profile and patient characteristics may shed light on a risk-stratified, therapeutic algorithm for RA patients.

## Supporting information

S1 Table Incidence of TB according to bDMARDs. (DOCX)

S2 Table Incidence of TB according to bDMARDs in patients without history of TB history. (DOCX)

S1 Dataset Supporting data set underlying the findings of this study. (XLSX)
