# Supplementary material for: The risk of tuberculosis disease in rheumatoid arthritis patients on biologics and targeted therapy: A 15-year real world experience in Taiwan
Source: PLoS One. 2017 Jun 1;12(6):e0178035. doi: 10.1371/journal.pone.0178035 (PMC5453436; doi:10.1371/journal.pone.0178035)
Supplement: S1 Table — (DOCX) [file pone.0178035.s001.docx]

Supporting Table 1. Incidence of TB according to bDMARDs

|  | **Total** | **Event (%)** | **Total**  **person-years** | **Incidence Rate (/10^5^ years)** | **IRR (95%CI)**† |
| --- | --- | --- | --- | --- | --- |
| **bDMARDs** |  |  |  |  |  |
| **ETN** | 443 | 13 (2.9) | 1461.8 | 889.3 | 1 |
| **ADA** | 332 | 11 (3.3) | 1042.1 | 1055.6 | 1.27 (0.76-2.13) |
| **GLN** | 60 | 0 (0.0) | 94.0 | 0 | - |
| **TCZ** | 31 | 0 (0.0) | 55.5 | 0 | - |
| **ABA** | 74 | 0 (0.0) | 105.3 | 0 | - |
| **TOF** | 11 | 0 (0.0) | 1.9 | 0 | - |
| **Total** | 951 | 24(2.5) | 2758.7 | 870.0 | - |

†Adjusted for sex and age

Abbreviations: TB, tuberculosis; bDMARDs, biological drug modifying anti-rheumatic drugs; CI, confidence interval; ETN, etanercept; ADA, adalimumab; GLN, golimumab; TCZ, tocilizumab; ABA, abatacept; TOF, tofacitinib; IRR, incidence rate ratio.
