# Supplementary material for: The risk of tuberculosis disease in rheumatoid arthritis patients on biologics and targeted therapy: A 15-year real world experience in Taiwan
Source: PLoS One. 2017 Jun 1;12(6):e0178035. doi: 10.1371/journal.pone.0178035 (PMC5453436; doi:10.1371/journal.pone.0178035)
Supplement: S2 Table — (DOCX) [file pone.0178035.s002.docx]

Supporting Table 2. Incidence of TB according to bDMARDs in patients without history of TB history

|  | **Total** | **Event (%)** | **Total person-years** | **Incidence Rate (/10^5^ years)** | **IRR (95%CI)**† |
| --- | --- | --- | --- | --- | --- |
| **bDMARDs** |  |  |  |  |  |
| **ETN** | 428 | 9 (2.1) | 1426.4 | 630.9 | 1 |
| **ADA** | 326 | 11 (3.4) | 1028.2 | 1069.9 | 1.87 (1.27-2.73) |
| **GLN** | 59 | 0 (0.0) | 92.5 | 0.0 | - |
| **TCZ** | 30 | 0 (0.0) | 54.5 | 0.0 | - |
| **ABA** | 70 | 0 (0.0) | 100.7 | 0.0 | - |
| **TOF** | 10 | 0 (0.0) | 1.7 | 0.0 | - |
| **Total** | 923 | 20(2.2) | 2703.9 | 739.7 | - |

†Adjusted for sex and age

Abbreviations: TB, tuberculosis; bDMARDs, biological drug modifying anti-rheumatic drugs; CI, confidence interval; ETN, etanercept; ADA, adalimumab; GLN, golimumab; TCZ, tocilizumab; ABA, abatacept; TOF, tofacitinib; IRR, incidence rate ratio.
